# Finite element analysis of 6 large PMMA skull reconstructions: A multi-criteria evaluation approach

**DOI:** 10.1371/journal.pone.0179325

**Published:** 2017-06-13

**Authors:** Angela Ridwan-Pramana, Petr Marcián, Libor Borák, Nathaniel Narra, Tymour Forouzanfar, Jan Wolff

**Affiliations:** 1 Department of Oral and Maxillofacial Surgery/Oral Pathology and 3D Innovationlab, VU University Medical Center, Amsterdam, The Netherlands; 2 Department of Maxillofacial Prosthodontics, Center for Special Care in Dentistry, Amsterdam, The Netherlands; 3 Institute of Solid Mechanics, Mechatronics and Biomechanics, Faculty of Mechanical Engineering, Brno University of Technology, Brno, Czech Republic; 4 BioMediTech Institute, Faculty of Biomedical Sciences and Engineering, Tampere University of Technology, Tampere, Finland; University of Zaragoza, SPAIN

## Abstract

In this study 6 pre-operative designs for PMMA based reconstructions of cranial defects were evaluated for their mechanical robustness using finite element modeling. Clinical experience and engineering principles were employed to create multiple plan options, which were subsequently computationally analyzed for mechanically relevant parameters under 50N loads: stress, strain and deformation in various components of the assembly. The factors assessed were: defect size, location and shape. The major variable in the cranioplasty assembly design was the arrangement of the fixation plates. An additional study variable introduced was the location of the 50N load within the implant area. It was found that in smaller defects, it was simpler to design a symmetric distribution of plates and under limited variability in load location it was possible to design an optimal for expected loads. However, for very large defects with complex shapes, the variability in the load locations introduces complications to the intuitive design of the optimal assembly. The study shows that it can be beneficial to incorporate multi design computational analyses to decide upon the most optimal plan for a clinical case.

## Introduction

Bone defects due to trauma or tumors are common in craniomaxillofacial (CMF) surgery. The reconstruction, hence cranioplasty, of such defects still remains a challenge. Cranioplasty primarily offers mechanical protection and subsequently seeks to restore the appearance of the patients. To date, the two most commonly used implant materials are polyetheretherketone (PEEK) and polymethylmethacrylate (PMMA). Furthermore, patient specific 3D printed titanium implants are being sporadically used [1]. Overall, PEEK and PMMA have similar properties. Both are biologically inert and maintain biomechanical properties similar to those found in bone [2]. PEEK is a synthetic material that has advantages in cranial-repair surgery, including strength, stiffness, durability, and inertness. PMMA on the other hand is easy to use, readily available and most importantly, biocompatible [3]. An advantage of PMMA is that it can be molded intra-operatively. Moreover PMMA is a relatively low cost option [4] with an extensive track record dating back to the 1940s.

Currently, cranioplasty treatment planning involves several steps. It starts with 3D tomographic imaging of the defect site, followed by computer modeling of the corresponding implant. After the implant is fabricated, the surgery is performed. During the surgery, the performing surgeon decides upon the fixation devices required and their subsequent arrangement based on his/her clinical experience and any peculiarities of the case.

In craniofacial surgery, stable fixation of bone is a prerequisite to achieving good results [5]. However, in spite of the best efforts, failures do occur due to a variety of factors [6]. Post operative infections are a major cause of reconstruction failures [7]. Such failures often occur due to tissue dehiscences commonly caused by a lack of a protective “watertight” soft tissue closure. Furthermore a reduced angiogenesis due to postoperative implant movement can jeopardize wound healing and tissue repair. Mechanical stability of cranial implants is pivotal for local vascularization and angiogenesis during bone regeneration. In an in-vivo study by Lienau et al [8], it was shown that smaller inter-fragmentary movements led to the formation of a greater number of vessels within the callus, particularly in areas close to the periosteum. While, larger movements increased inter-fragmentary shear and reduced vascularisation during early bone healing. Reduced angiogenesis due to postoperative implant movement can jeopardize wound healing and tissue repair, and can subsequently lead to complications after reconstructive surgery. Besides biological factors, several design factors influence the quality and stability of the implant assembly; to name a few, shape, fixation device arrangement and osteotomy geometry [9]. Another important aspect to take into account when designing an implant is its load-bearing capacity. It is evident that the outcome of cranial reconstruction is markedly influenced by a wide range of factors, in addition to the surgeons skills and experience. In the case of large defects, the surgeons experience becomes especially critical and yet may not be sufficient to give enough consideration to the numerous variables that may contribute to the long term success. The surgeon may design multiple treatment options for a particular case, but the best option may not be intuitively apparent. In such cases, it may be beneficial to have a quantitative assessment of these options based on established bio-mechanical paradigms. In this regard, numerical simulations have become an important method in biomechanics field since they allow to estimate the load-bearing capacity of the design without the need of making a prototype and performing a mechanical test [10].

With increasing incorporation of technology and investment in computational infrastructure at clinical institutions, it is reasonable to pursue the most optimal treatment option for customized skull reconstructions. The feasibility and availability of medical grade rapid fabrication further enhances the prospects for a composite planning approach. This study analyses 6 clinical cases in terms of skull defect size, incident load locations and fixation device arrangement. With increasing size and/or shape complexity of the defect, the possible combinations of fixation arrangement and loading grows and consequently the number of options for the implant assembly increases significantly. A metric to evaluate these options in terms of relative performance of relevant biomechanical parameters in components (stress, strain etc.), is presented in this study.

## Materials and methods

### Construction of computational models

**Fig 1:**
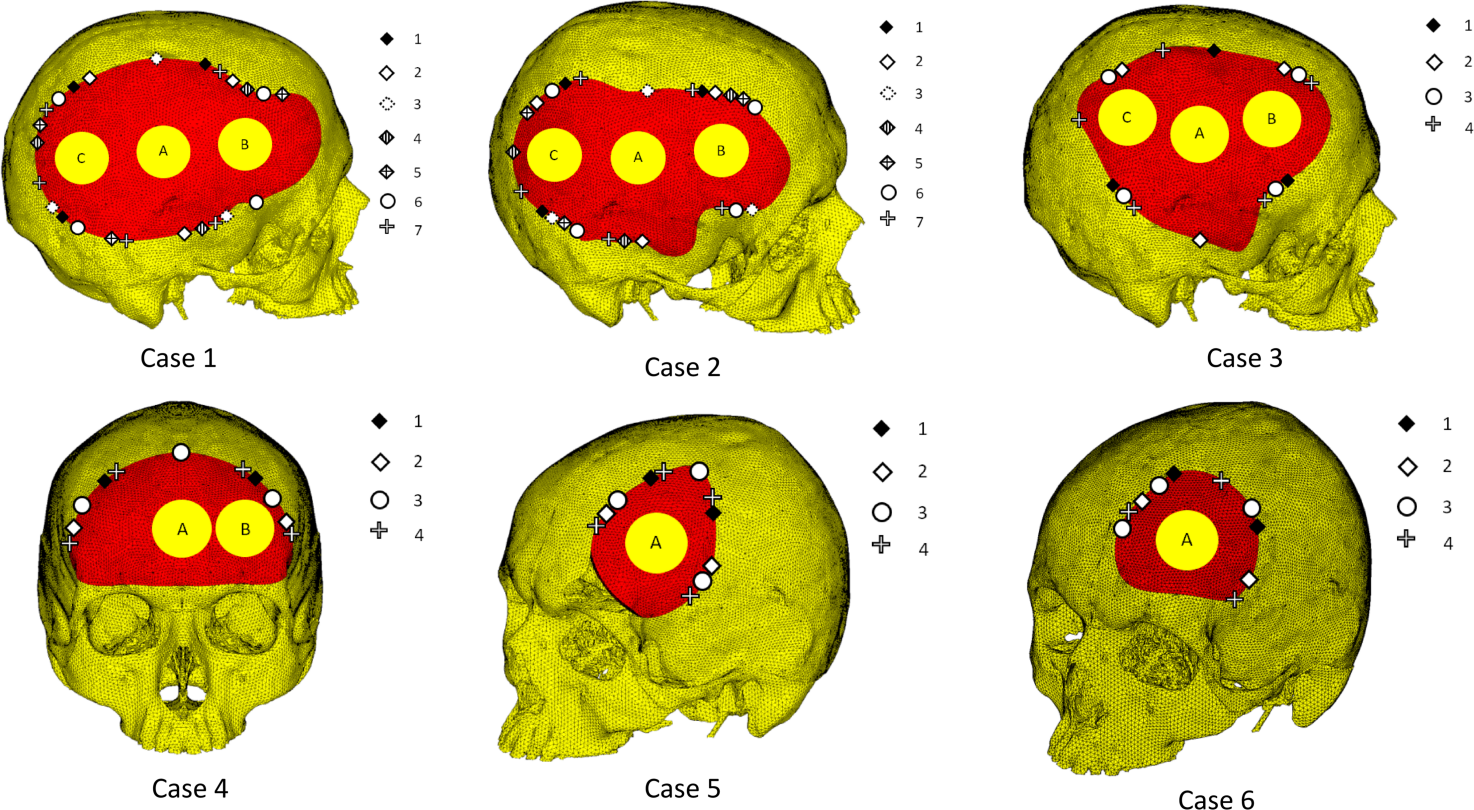
Skull reconstruction plans modeled for 6 cases with large defects. Models for each case illustrate the implant shape and location (red); fixation device numbers and arrangement (markers at implant boundaries); and loading locations for incident 50N static loads (labeled circular patches: A, B or C). Of note, the different layouts of the fixations devices on the periphery of the implant—in terms of numbers and position—are illustrated in corresponding marker styles. These layouts are assigned an identifying number indicated in the adjacent legend for each case.

The computational models constructed in this study represent a system of 4 components: PMMA implant, skull and plates with screws (fixation devices). For each of the 6 clinical cases a generic model of skull was used. The generic model was reconstructed from a CT dataset obtained from clinical Case 2. The CT image volume consisted of 264 cross-sectional slices with pixel dimensions of 0.7 × 0.7 mm and slice thickness of 1.0 mm. This case was the most suitable for simulating all examined defects of Cases 1 through 6. The CT dataset was imported into an image processing software (STL Model Creator [11,12]) programmed by the authors in a numerical computing environment (Matlab 2010, MathWorks, Natick, MA, USA) to construct the 3D polygonal stereolitography (STL) model of the skull. The segmentation of the skull bone as one homogeneous compartment was performed through greyscale thresholding of the CT images. The PMMA implants were individually designed based on the defect shape for each patient. The curvature of the implant was decided upon by assuming the patient’s skull to be symmetrical along its mid-sagittal plane. This facilitated the replication of the missing bone fragment from a mirrored volume of the contra-lateral side of the skull. PMMA implant and skull were modeled in 3D in SolidWorks (Dassault Systems, Vélizy-Villacoublay, France). The 3D geometries of the individual components were assembled in Ansys 17.2 (Swanson Analysis Systems Inc., Houston, PA, USA) and finite element meshes were subsequently constructed. All volumes were discretized using a higher-order, 3-D hexahedral, 10-node element type (SOLID187). In a typical surgical procedure, fixation devices (plates and screws) are used to affix PMMA implants to the skull. A thorough investigation of mechanical competence of an assembly would consequently require the fixation device to be included in the model. To avoid increasing the complexity of the model, the fixation devices were modeled implicitly by adding an equivalent stiffness to the global FE stiffness matrix of the skull and PMMA plate. The equivalent stiffness was applied by using an element type with undefined geometry but with specified additional stiffness coefficients in a matrix form (MATRIX27). The stiffness coefficients were calculated in a previous study [9] and are listed in Table 1. All interfaces between components were connected using elements representing 3 dimensional contact and sliding between the target surfaces and a deformable contact surface (TARGE160 and CONTA174). A conservative friction coefficient of 0 was used for all contact pairs. All participating volumes were modeled using linear, homogeneous, and elastic material type and a corresponding Young’s moduli and Poisson’s ratios were used as listed in Table 2. The values listed are not specific to the particular components used in the reconstruction. They are instead representative values for the material taken from literature. The model of the implant assembly is evaluated under loading conditions reflecting a scenario where the head of the patient rests on a hard surface. The point of contact is modelled to be incident over a small area on the surface of the implant. Loading of the skull and PMMA implant is assumed to be equivalent to the weight of the head itself. Specifically, a weight of 5 kg [13] is assumed based on simplified measurements on small number of specimens. This load was applied to the model as a pressure force of 50N distributed over a small area as indicated in Fig 1. The global boundary conditions were held constant across all configurations by fixing the skull base in the proximity of the spinal region [14].

**Table 1:** Stiffness coefficients used in representing fixation devices.

	Stiffness	units
Tension	2000	N/mm
Torsion	153.85	Nmm/rad
Strong axis bending	399.8	N/mm
Strong axis shear	16.87	N/mm
Weak axis bending	73.04	N/mm
Weak axis shear	0.49	N/mm

**Table 2:** Material properties.

	Bone	PMMA
**Young's Modulus**	E_b_ = 15000 MPa	E_PMMA_ = 3000 MPa
**Poisson's Ratio**	μ_b_ = 0.3	μ_PMMA_ = 0.38
**Yield strength**	-	σ_y,PMMA_ = 65 MPa
**References**	[15,16]	[17–19]

### Model configurations

Each of the 6 cases were modeled and simulated individually. The arrangement of the fixation plates and the location of the external load (50N) for each case were determined by the chief surgeon according to the requirements for corresponding treatment. The surgeon’s recommendations were designated as model configurations for each case (total 70 configurations). For the sake of brevity, these configurations are presented in Fig 1 through a scheme. The defect cases large enough to make variation of load location possible have their respective positions indicated by alphabetized patches. The number and arrangement of the fixation devices are illustrated through unique markers (for a case). To be able to identify these configurations in this work, they were systematically labeled using a three-character code. The first character in the code (a number) denotes the specific case, the second character (a letter) denotes the point of loading, and the third character (a number) denotes the fixation device arrangement. For instance, label “4B2” denotes Case 4, Load B and fixation arrangement 2 (scheme defined in Fig 1). The element/node counts for the 6 models were around 850k/1100k and the configuration details are described below:

#### Case 1

Seven variations in the titanium plate position and three variations in load sites were assessed. In arrangements "1" through "5", 3 titanium plates were used. In arrangements "6" and "7", 4 and 5 titanium plates were used, respectively. All 21 configurations analyzed in this case are labeled as follows: 1A1-1A7, 1B1-1B7, and 1C1-1C7.

#### Case 2

In this case, seven variants of titanium plate position and three variants of load were assessed. In configurations "1" through "5", 3 titanium plates were used. In configurations "6" and "7", 4 and 5 titanium plates were used, respectively. The 21 configurations analyzed in this case are labeled as follows: 2A1-2A7, 2B1-2B7, 2C1-2C7.

#### Case 3

In this case, four variants of titanium plate position and three variants of load were used. In configurations "1" and "2", 3 titanium plates were used. In configurations "3" and "4", 4 and 5 titanium plates were used, respectively. The 12 configurations analyzed in this case are labeled 3A1-3A4, 3B1-3B4, 3C1-3C4.

#### Case 4

In this case, four variants of titanium plate position and two variants of load were used. In configurations "1" and "2", 2 titanium plates were used. In configurations "3" and "4", 3 and 4 titanium plates were used, respectively. 8 configurations analyzed in this case are labeled 4A1-4A4, 4B1-4B4.

#### Case 5

In this case, four variants of titanium plate positions and a single loading condition were used. In configurations "1" and "2", 2 titanium plates were used. In configurations "3" and "4", 3 and 4 titanium plates were used. 4 configurations analyzed in this case are labeled 5A1-5A4.

#### Case 6

In this case, four variants of titanium plate position and a single loading condition were used. In configurations "1" and "2", 2 titanium plates were used. In configurations "3" and "4", 3 titanium plates were used. 4 configurations analyzed in this case are labeled 6A1-6A4.

### Configuration assessment

The performance of each configuration was analyzed in terms of stresses and strains induced in each component of the assembly. Stress intensity and displacement in the PMMA implant, shear/normal forces in fixation plates, and the induced strain in the bone are important parameters that could affect the overall clinical outcome. Therefore a metric, called assessment factor (AF), was formulated as a weighted combination of the aforementioned parameters. This factor was based on a multi-criteria decision-making (MCDM) approach known as Weighted Sum Method (WSM) [20]. This method is commonly used and widely popular in the field of computational mechanics.

For an analysis of M alternatives (A_i_ for i = 1, 2, … M) and N criteria (C_j_ for j = 1, 2, … N), the favorableness of the i-th alternative can be calculated by the following expression:

$$AF_{i}=\sum_{j=1}^{N}w_{j}\cdot y_{ij}$$ (1)

Where, AF_i_ is the WSM-score or the assessment factor of the i-th alternative, y_ij_ is the value of the i-th alternative with respect to the j-th criterion and w_j_ is the weighting factor of the j-th criterion. The latter expresses the importance of C_j_. Throughout this study, the alternatives are all configurations within each case; e.g. for the Case 1, the alternatives are A = {1A1, 1A2, 1A3, 1A4… 1C5, 1C6, 1C7}. The criteria evaluated here are C = {SINT, EPTO, UNORM, FNORM, FSHEAR}, where

SINT = stress intensity in the PMMA [MPa]

EPTO = strain intensity in the bone [–]

UNORM = total normal displacement (normal to brain surface) of PMMA plate [mm]

FNORM = normal force in the titanium plate [N]

FSHEAR = total shear force in the titanium plate [N]

These parameters represented nominal values in specific regions of interest. These values were evaluated to represent a typical biomechanical condition of the case/configuration and to be a basis for a comparative analysis (S1 File, S1 Table). Since these parameters have different dimensions and different units, Eq 1 cannot be used directly and the source data must be modified (normalized) first. Assuming a linear dependency between the normalized values and the actual values, 0 is assigned to the worst value of C_j_ and 1 is assigned to the best value of C_j_. Therefore, the normalized values can be calculated as follows:

$$u_{ij}=\frac{y_{ij}-d_{j}}{h_{j}-d_{j}}$$ (2)

Where, d_j_ and h_j_ are maximum and minimum values of C_j_, respectively. Finally, the assessment factor for the normalized values can be calculated using following expression:

$$AF_{i}=\sum_{j=1}^{N}w_{j}\cdot u_{ij}$$ (3)

The best alternative is the one that satisfies following expression:

$$AF_{BEST}=\max\left(AF_{i}\right),\;for\;i=1,2,3,\ldots ,M$$ (4)

On the contrary, the least favorable alternative is the one that minimizes the assessment factor as follows:

$$AF_{WORST}=\min\left(AF_{i}\right),\;for\;i=1,2,3,\ldots ,M$$ (5)

The importance of the individual criteria plays a significant role and the weighting factors must be chosen carefully. In doing so, their sum must equal 1. In case of uncertainty or in the case that all criteria are assumed to be equally important (as is the case in the present study), the weighing factors can be calculated as follows:

$$w_{j}=\frac{1}{N}$$ (6)

Therefore, in this study the weighting factors are 0.2 for all evaluated criteria. For the final comparison, the assessment factors within each case were additionally normalized by mean value of all AFs and their standard deviation using following expression:

$$AF_{i}^{*}=\frac{AF_{i}-\mu }{\sigma }$$ (7)

Where, μ is the mean value of assessment factors within the assessed case and σ is the standard deviation of assessment factors within the assessed case. This adjustment allows the best configuration to be presented with a positive AF and, the worst configurations with negative AF.

The assumption of weighting factors equality for all evaluated quantities may be debatable. Choice of different weights might lead to determining of different best/worst configuration within the case. In order to verify the best/worst configuration choice, a testing study within the WSM was carried out using all possible non-trivial permutations of participating criteria. Since five parameters were evaluated and taken as a basis for MCDM, a total of 2^5^–1 = 31 non-trivial permutations were tested for the best/worst configurations. In other words, the configurations were evaluated firstly based on only one parameter (i.e. individually SINT, EPTO, UNORM, FNORM, FSHEAR), secondly based on combinations of two parameters (SINT+EPTO, SINT+UNORM….), etc., and finally based on the combination of all parameters. Frequencies of best/worst combinations throughout all non-trivial permutations were determined. The most frequent best/worst combination was assumed to be the solution of this verification study.

## Results

**Fig 2:**
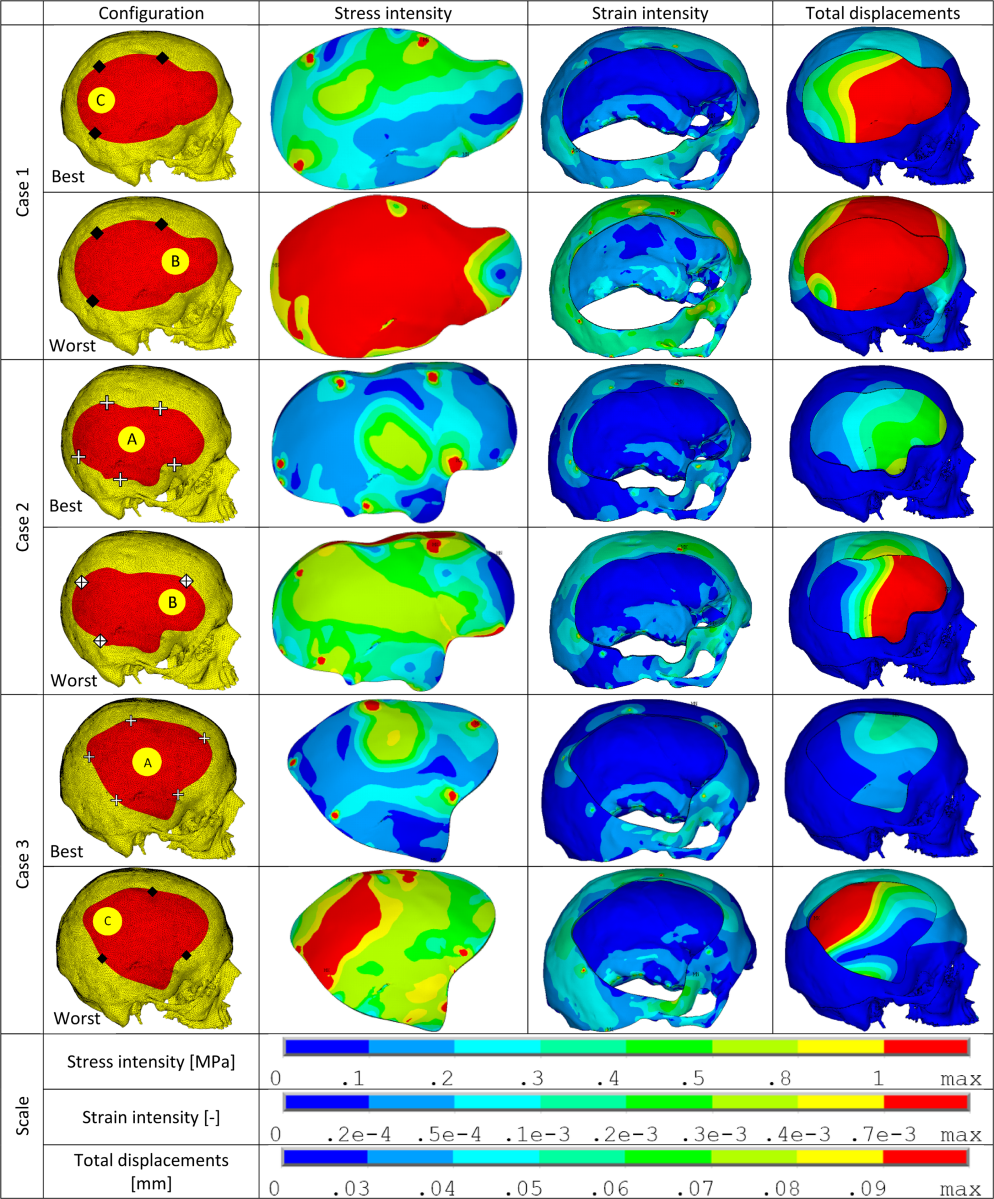
Stress intensity, strain intensity and displacement plots of best/worst performers within each case (cases 1 through 3). The configuration details are illustrated using the marking scheme introduced in Fig 1. In the illustrations–stress intensity is in PMMA implant, strain intensity is in the bone and the total displacements are for the PMMA implant.

**Fig 3:**
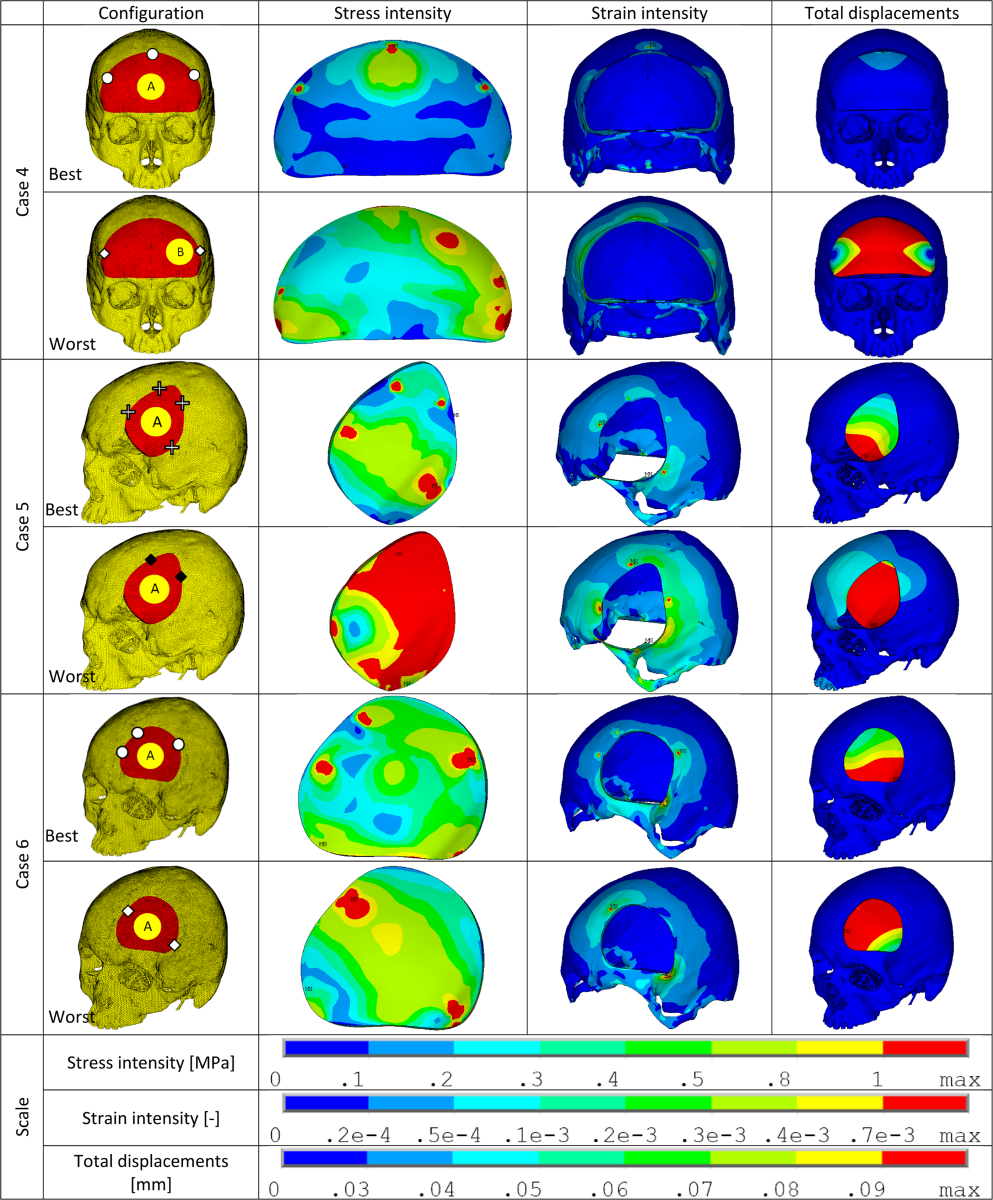
Stress intensity, strain intensity and displacement plots of best/worst performers within each case (cases 4 through 6). The configuration details are illustrated using the marking scheme introduced in Fig 1. In the illustrations–stress intensity is in PMMA implant, strain intensity is in the bone and the total displacements are for the PMMA implant.

**Fig 4:**
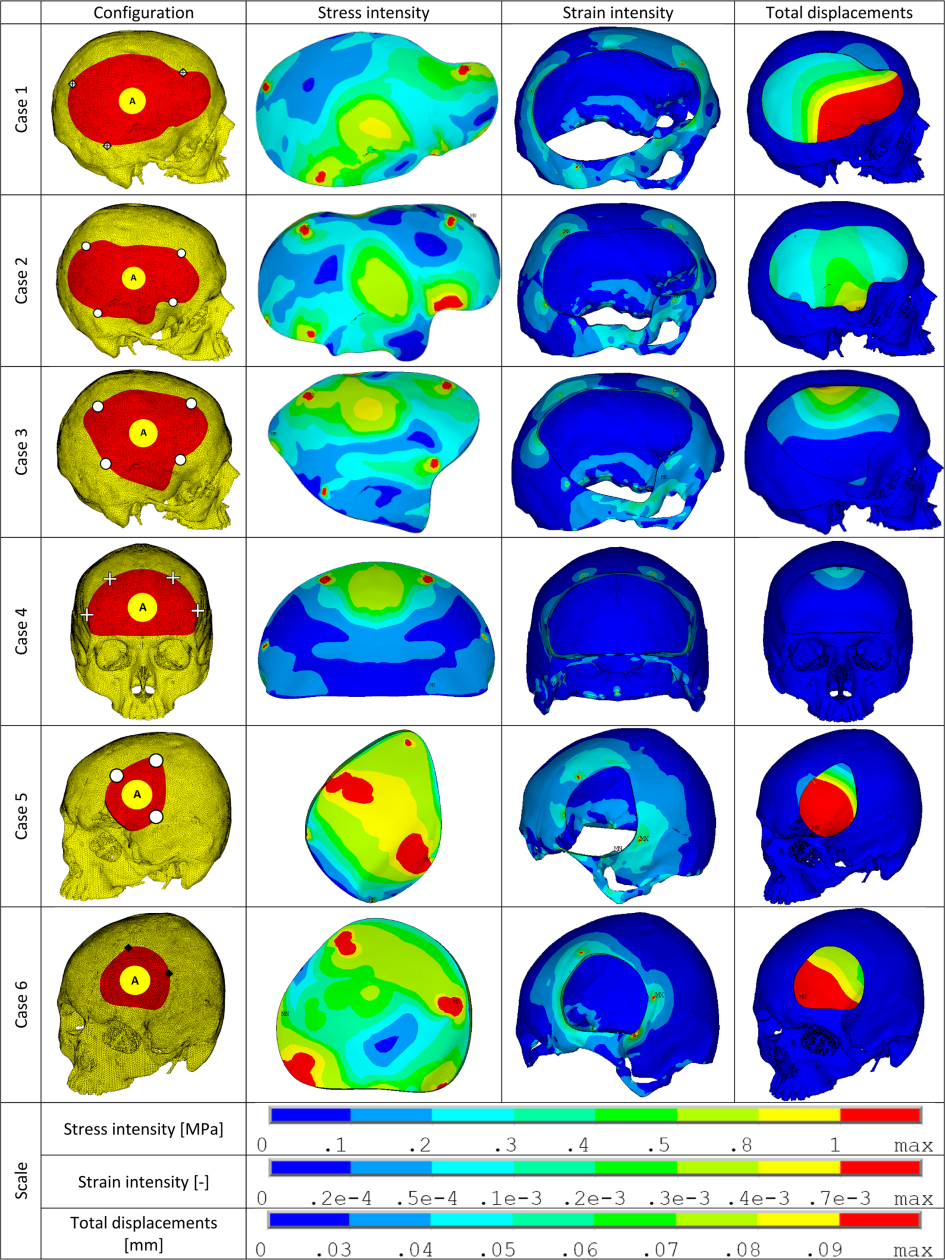
Stress intensity, strain intensity and displacement plots of second-best performers within each case (cases 1 through 6). The configuration details are illustrated using the marking scheme introduced in Fig 1. In the illustrations–stress intensity is in PMMA implant, strain intensity is in the bone and the total displacements are for the PMMA implant.

The various configurations for each case (Case 1 through Case 6) were simulated under static loading conditions and the results were analyzed for the following mechanically relevant parameters–total displacements (PMMA implant), stress intensity (PMMA implant), strain intensity (bone), and normal and shear forces (fixation plate). The values obtained were used to calculate the assessment factors. The best and worst case configurations based on this factor are illustrated in Fig 2 (cases 1, 2 & 3) and Fig 3 (cases 4, 5 & 6). The configurations with the second best performance in each case are illustrated separately in Fig 4. In the following sub-sections, the extreme values of the evaluated parameters are listed.

### Case 1

**Fig 5:**
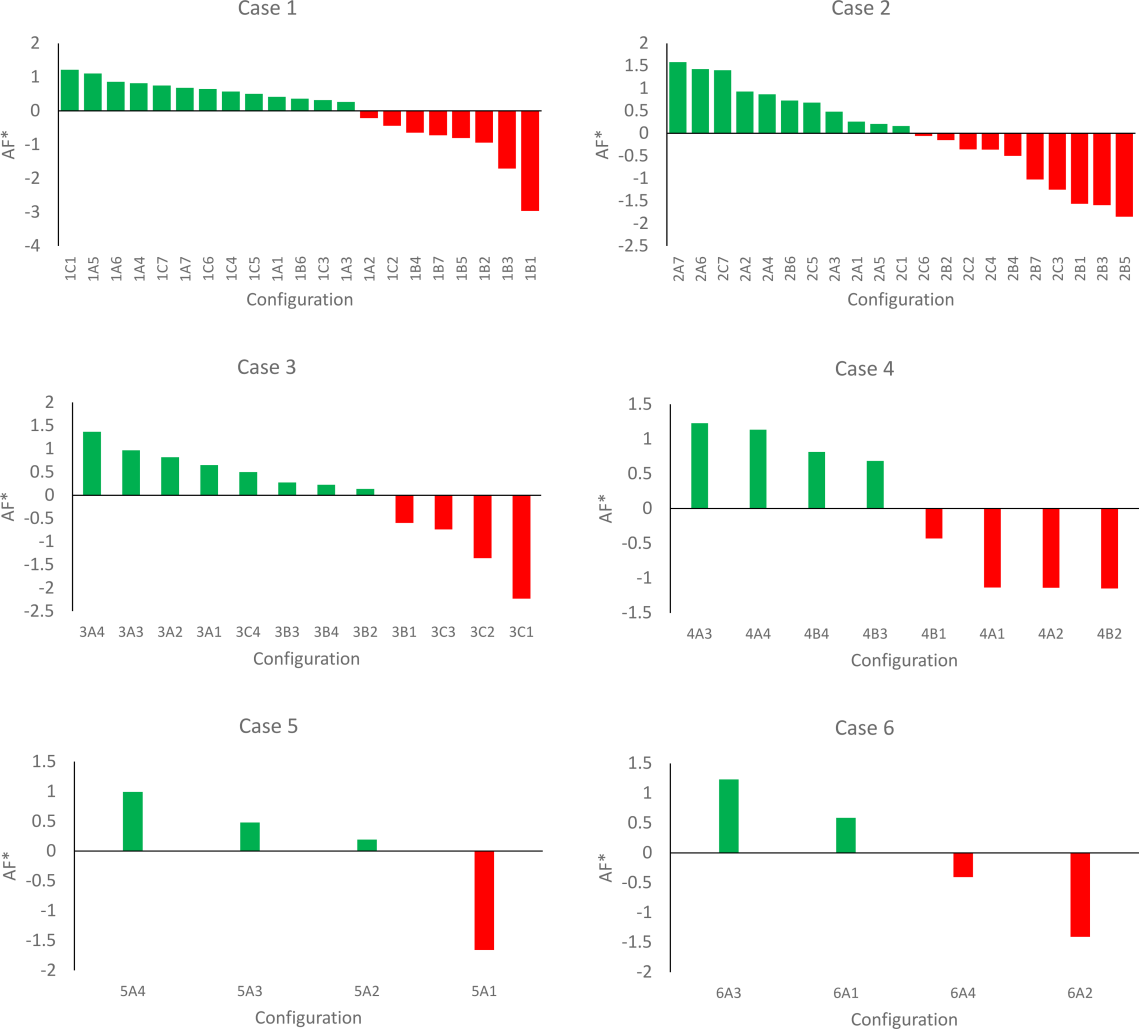
Normalized assessment factors (AF*) for every configuration in each case. The configurations are sorted by magnitude of AF* (i.e. higher values indicate better performance). Configuration ID on the horizontal axis can be deciphered through Fig 1.

**Fig 6:**
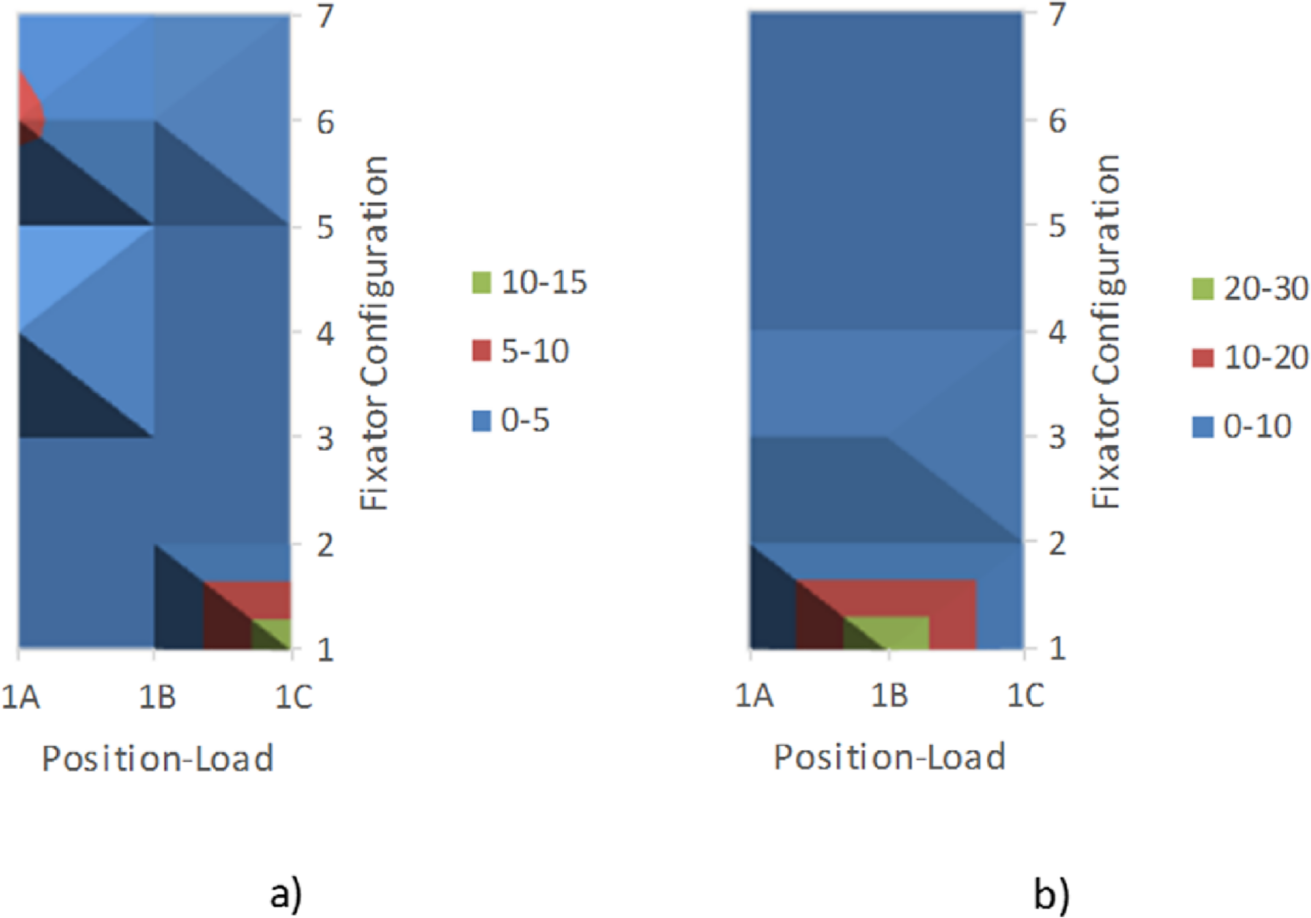
Example of the verification test based on a permutation method. Frequency of best/worst configuration determined for all non-trivial permutations: a) Case 1, Best configuration, b) Case 1, Worst configuration.

Among the 21 configurations, the maximum values observed for each parameter were–displacement: 1.26 mm (1B1); stress intensity: 11.45 MPa (1A3); strain intensity: 1.91x10^-3^ (1B1); normal force: 53.29 N (1B1); shear force: 19.83 N (1B1). The calculated assessment factor AF_i_^*^ for each configuration is illustrated in Fig 5. 1B1 was the worst performer and 1C1 was the best performer. Fig 2 illustrates stress, strain and displacement plots for the best/worst configurations. Fig 6 shows a typical example of the verification study based on the permutation approach. The results show that the best/worst configurations for this case as presented above apply for most of analyzed permutations of weighting factors in consideration.

### Case 2

Among the 21 configurations, the maximum values observed for each parameter were–displacement: 0.32 mm (2B5); stress intensity: 5.07 MPa (2A2); strain intensity: 1.41x10^-3^ (2B7); normal force: 51.14 N (2B1); shear force: 24.02 N (2C3). The calculated assessment factor AF_i_^*^ for each configuration is illustrated in Fig 5. 2B5 was the worst performer and 2A7 was the best performer. Fig 2 illustrates stress, strain and displacement plots for the best/worst configurations.

### Case 3

Among the 12 configurations, the maximum values observed for each parameter were–displacement: 0.37 mm (3C1); stress intensity: 5.08 MPa (3C1); strain intensity: 1.30x10^-3^ (3C1); normal force: 36.62 N (3B2); shear force: 10.76 N (3C2). The calculated assessment factor AF_i_^*^ for each configuration is illustrated in Fig 5. 3C1 was the worst performer and 3A4 was the best performer. Fig 2 illustrates stress, strain and displacement plots for the best/worst configurations.

### Case 4

In the 8 configurations, the following were the observed maximum values–displacement: 0.34 mm (4A2); stress intensity: 7.14 MPa (4A2); strain intensity: 1.08x10^-3^ (4A1); normal force: 24.53 N (4B1); shear force: 3.52 N (4A1). In this case, only two loading cases were analyzed (because of symmetry—the results would be same). The calculated assessment factor AF_i_^*^ for each configuration is illustrated in Fig 5. 4B2 was the worst performer and 4A3 was the best performer. Fig 3 illustrates stress, strain and displacement plots for the best/worst configurations.

### Case 5

In the 4 configurations, the following were the observed maximum values–displacement: 1.11 mm (5A1); stress intensity: 10.91 MPa (5A1); strain intensity: 1.85x10^-3^ (5A1); normal force: 44.43 N (5A1); shear force: 39.44 N (5A1). The calculated assessment factor AF_i_^*^ for each configuration is illustrated in Fig 5. 5A1 was the worst performer and 5A4 was the best performer. Fig 3 illustrates stress, strain and displacement plots for the best/worst configurations.

### Case 6

In the 4 configurations, the following were the observed maximum values–displacement: 0.15 mm (6A1); stress intensity: 4.25 MPa (6A4); strain intensity: 1.58x10^-3^ (6A4); normal force: 40.99 N (6A3); shear force: 9.94 N (6A2). The calculated assessment factor AF_i_^*^ for each configuration is illustrated in Fig 5. 6A2 was the worst performer and 6A3 was the best performer. Fig 3 illustrates stress, strain and displacement plots for the best/worst configurations.

## Discussion

In cranioplasty the mechanical robustness of the implant assembly is of paramount importance for long-term clinical success. In this finite element study of clinical cases, 6 patient specific PMMA-based skull reconstruction assemblies for large skull defects were evaluated under 50N static loads. In a recent finite element study of skull implants [9], shape, fixation device arrangement and osteotomie geometry were shown to influence the stress and strain distribution. These biomechanical insights were combined with the performing surgeon’s clinical experience and incorporated in the reconstruction plans. Multiple configurations for every patient were designed and assessed through the WSM based assessment factor.

In order to evaluate the performance of every configuration, their mechanical response in terms of strains, stresses and forces within the bodies of the individual components were observed (PMMA implant, fixation device and bone). In all configurations, the implant displacements were largely in the sub-millimeter domain. Only two configurations showed large displacements of 1.11 mm (5A1) and 1.26 mm (1B1). The largest stresses were observed in the PMMA implant body in the range of 5–10 MPa, which is well below the material yield (failure) stress of 65 MPa. A significant area of concern can be the anchoring capabilities of the fixation screws. Besides the inherent variability in bone quality, significantly high strains at the screw-bone interface can induce a failure in the local structural integrity of bone; subsequently compromising the stability of the entire assembly. The results indicate that the maximum strains (max: 1500 με) are well below the assumed unphysiological limit of 3000 με [21]. In keeping with the scope of the study, the simulation results represent nominal values that are sufficient for comparative optimality analyses. Thus the values should be interpreted with care and not taken to represent an accurate assessment of strength of the components. Such an assessment of the configurations would necessitate detailed modeling and analysis, especially at the component interfaces. On the other hand, the modeling and simulation setup of this study showcases an effective method in relative evaluation of multiple design options, while avoiding extremely large and complex model setup, and proportional computational expense.

The 6 cases studied represent a distribution of defect sizes. The complexity and number of available options for a case increases with the size of the defect. In reconstructions with smaller defects (cases 5 & 6), the number of combinations of fixation device arrangement and loading points is relatively low. That is, with lower variations in the incident loading points, it becomes easier to design an optimal fixation device arrangement. Consequently, distinguishing between configurations in terms of performance (through the assessment factor) is a simpler task (Fig 5). However, in cases with large defects the point of incidence of loading can vary greatly and the fixation device arrangement will have to accommodate every such possibility. Thus by using the assessment factor it becomes possible to not only grade the configurations with respect to relative performance, but equally significantly, it is possible to see patterns of performance. For example, in case 1 (Fig 5) it can be seen that configurations with loading B seem to consistently perform poorly. Similarly in Case 2 (Fig 5), configurations with loading A seem to perform better than B or C. Thus where needed the fixation device arrangement can be modified to adjust performance accordingly (i.e. improve loading B in case1). Alternatively, if it can be ascertained that a particular loading point is either unlikely or the degraded performance acceptable, then fixation devices can be re-arranged to improve performance for other loading points. From a cursory look at the results it can be gathered that the area of incidence of an expected load and the arrangement of fixation devices with respect to it is an important factor. The results tend to be favorable when the loading area lies within the convex hull formed by the fixation device positions. However, on examining the results in detail, this trait may be too simplistic for larger defects and may only serve as a good starting point towards designing the best option. Large defect geometries can be complex enough to induce unforeseen behavior under loading at particular locations. That is, when the loading is asymmetric with respect to the fixation devices (outside convex hull), the interface between the implant and bone plays a significant role. Additionally, the location of these defects in the anatomy and the criticality of the surrounding anatomy can be very important factors. Thus, a large defect on the forehead may have a different optimum when compared to the same defect geometry at the back of the skull. The size and extent of the defect itself can lead to a reduction in skull rigidity, affecting design considerations. The results for worst case configurations in Fig 2 indicate a trend where the displacement of the implant is accompanied by noticeable displacement of the surrounding bone. The largest bone displacement among all cases is observed in configuration 1B1 (0.17mm). In contrast, the worst cases in the comparatively smaller defects in Fig 3 do not show similar magnitudes of bone displacement. In configuration 1B1 the increased deformability of the bone on top of the skull (due to loss of rigidity) in combination with the transfer of loads through the solitary fixation plate in close proximity to the applied load, results in large displacement in the adjacent bone. Apart from the loss of rigidity due to the defect size and location, factors such as skull thickness (adjacent to defect), bone material quality and load transfer between the implant and bone can further influence its rigidity under loading. This serves to reinforce the efficacy of a comparative computational approach in deciding between treatment options.

While we only investigated the implant assemblies with PMMA material, other materials (e.g. PEEK) with similar properties will behave mechanically similarly. However more rigid materials such as titanium alloys may alter design parameters considerations as their rigidity affects the transfer of stresses/strains among the fixation devices (screws and plates) and bone. Tsouknidas et al [22] compared PMMA and Ti-alloy cranial implants in a FEM study. Even though PMMA cannot withstand forces endured by Ti alloys, its mechanical strength is sufficient to tolerate higher loads than the vicinal bone tissue thus providing sufficient neurocranial protection in terms of fracture strength [22].

The assessment factor used in this study can be further tuned by weighting different parameters in order of their sensitivity or, alternatively, other MCDM methods might be used (Weighted Product Method, Analytic Hierarchy Process etc.). The weighting of parameters may also be tuned with regard to the specific requirements of the analysis. For example, stresses in implant body can be nonlinearly weighted such that, as long as it is below a critical material failure limit, other parameters can be weighted more. Alternatively, strains in the bone can be given weights empirically designed to minimize bone degradation due to unphysiological strains (too little or too much can result in bone degradation). In this study the variables that are involved in designing a reconstruction assembly have been limited to fixation plate number, arrangement and loading location. There are however many more that can dictate the final design such as: osteotomy angulation, bone quality, aesthetic considerations, proximal critical anatomy etc. In the designed configurations of this study, the chosen fixation arrangements are only a subset of all the possible options. The best and second-best configurations indicate that the precise location for a single plate is not unique for optimal (or acceptable) performance. Ideally the fixation plates work together to equally stabilize the implant under loads, thus creating a dependency among the plates. For a given loading scenario, if the relative placement of the plates is consistent, the precise location or orientation of the arrangement can be flexible. That is to say, there can be multiple ways to position the plates such that the incident load is distributed among the plates to make them perform similarly. In a clinical scenario however, the presence of the aforementioned design factors (bone quality, aesthetic considerations, proximal critical anatomy) limit this flexibility. Thus the design and evaluation of the treatment options should aim for acceptable performance through quantitative assessment rather than finding the absolute single best design. Consequently, this study exhibits the utility of computational analysis and multi-factor based assessment in evaluating multiple treatment options for very large skull defect reconstructions.

## Conclusion

From an engineering perspective, a cranioplasty reconstruction design should aim to minimize the possibility for extreme strains/stresses in the assembly under normal operating conditions, while predicting and optimizing performance for unexpected incidents (such as impacts on the cranium due to minor accidents or falls). This involves, on the one hand, accounting for expected loads (magnitude and direction) on the implant assembly, and on the other, building a safety factor into the assembly design (in terms of arrangement and numbers of: plates and screws) while taking into account natural variables such as osteotomy geometry, defect size/shape/location. It takes further significance in presence of clinical restrictions that limit design choices such as proximity to critical anatomies. With increasing defect size the complexity of the design increases, along with the requirement for mechanical stability and aesthetic conformity. Thus it is advantageous to use computational tools to reach the most effective solution with reasonable pre-operative effort which would minimize the intra and post-operative effort. In this study we show that FEA based computational analysis can be used to give surgeons an opportunity to evaluate multiple treatment options. A range of assembly combinations, from the minimalist to the conservative, can be assessed through the procedure employed in this work. Thus, ‘best practices’ in design can be combined to reach an optimal solution within the constraints of a particular clinical case. This could help the surgeon to pre-operatively plan the design of the implant including the fixation plates.

## Supporting information

S1 File Raw data from FEA calculations representing nominal values in specific regions of interest. (ZIP)

S1 Table Sorted data from FEA calculations representing nominal values in specific regions of interest. (XLSX)
